# Phytochemicals: Potential Therapeutic Interventions Against Coronavirus-Associated Lung Injury

**DOI:** 10.3389/fphar.2020.588467

**Published:** 2020-11-18

**Authors:** Mohammad Bagher Majnooni, Sajad Fakhri, Yalda Shokoohinia, Narges Kiyani, Katrina Stage, Pantea Mohammadi, Mohammad Mehdi Gravandi, Mohammad Hosein Farzaei, Javier Echeverría

**Affiliations:** ^1^ Student Research Committee, Kermanshah University of Medical Sciences, Kermanshah, Iran; ^2^ Pharmaceutical Sciences Research Center, Health Technology Institute, Kermanshah University of Medical Sciences, Kermanshah, Iran; ^3^ Ric Scalzo Botanical Research Institute, Southwest College of Naturopathic Medicine, Tempe, AZ, United States; ^4^ Medical Biology Research Center, Health Technology Institute, Kermanshah University of Medical Sciences, Kermanshah, Iran; ^5^ Departamento De Ciencias Del Ambiente, Facultad De Química y Biología, Universidad De Santiago De Chile, Santiago, Chile

**Keywords:** coronaviruses, lung injury, phytochemicals, COVID-19, signaling pathway

## Abstract

Since the outbreak of coronavirus disease 2019 (COVID-19) in December 2019, millions of people have been infected and died worldwide. However, no drug has been approved for the treatment of this disease and its complications, which urges the need for finding novel therapeutic agents to combat. Among the complications due to COVID-19, lung injury has attained special attention. Besides, phytochemicals have shown prominent anti-inflammatory effects and thus possess significant effects in reducing lung injury caused by severe acute respiratory syndrome coronavirus 2 (SARS-CoV-2). Also, the prevailing evidence reveales the antiviral effects of those phytochemicals, including anti-SARS-CoV activity, which could pave the road in providing suitable lead compounds in the treatment of COVID-19. In the present study, candidate phytochemicals and related mechanisms of action have been shown in the treatment/protection of lung injuries induced by various methods. In terms of pharmacological mechanism, phytochemicals have shown potential inhibitory effects on inflammatory and oxidative pathways/mediators, involved in the pathogenesis of lung injury during COVID-19 infection. Also, a brief overview of phytochemicals with anti-SARS-CoV-2 compounds has been presented.

## Introduction

The complex pathophysiological mechanisms behind viral diseases, along with the associated side effects of the present conventional drugs, urge the need for introducing alternative treatments. Among viral infections, severe acute respiratory syndrome coronavirus (SARS-CoV), Middle East respiratory syndrome (MERS-CoV), and the newest human CoVs (HCoVs) associated with the outbreak of coronavirus disease 2019-SARS-CoV-2 (COVID-19) have caused acute respiratory distress syndrome (Sharma et al., 2020). Based on the pathological findings, the inflammatory cytokines/signaling pathways lead to pulmonary edema and, ultimately, lung injury in COVID-19 patients (Merad and Martin, 2020). Considering their potential effects in targeting several dysregulated mediators, phytochemicals could be auspicious agents in the treatment/management of various diseases (Mani et al., 2020). The medicinal plants and phytochemicals target multiple proinflammatory and oxidative mediators such as tumor necrosis factor-α (TNF-α), interleukin- (IL-) 1β, IL-6, IL-8, matrix metalloproteinases (MMPs), nuclear factor-kappa B (NF-κB), mitogen-activated protein kinase (MAPK), cyclooxygenase-2 (COX-2), inducible nitric oxide synthase (iNOS), and reactive oxygen species (ROS). Therefore, owing to the involvement of inflammation and oxidative stress in the pathogenesis of lung injury, phytochemicals have attracted particular attention to providing novel agents in combating coronaviruses and related complications (Bellik et al., 2012; Cornélio Favarin et al., 2013b). This article presents an overview of phytochemicals, including alkaloids, coumarins, polyphenols, especially flavonoids, quinones, and terpenes to show noticeable effects against lung injury. Therefore, they could be introduced as ameliorative agents against SARS-CoV-2-induced lung injury. Moreover, based on their simultaneous antiviral and preventive effects against lung injury, some phytochemicals such as matrine, cepharanthine, osthole, wogonin, myricetin, and triptolide have been also provided as promising candidates in the management of COVID-19. In general, this review article aims to introduce phytochemicals as potential therapeutic agents against coronavirus complications, focusing on lung injury.

## Coronaviruses and Pathogenesis: Focusing on Lung Injury

In striking contrast to the history of HCoVs, as relatively harmless respiratory pathogens, the outbreak of SARS and the emergence of MERS pose the CoVs as important pathogens in respiratory tract infections. SARS-CoV, MERS-CoV, and SARS-CoV-2 can cause clinical complications leading to severe diseases presented as acute respiratory distress syndrome (ARDS) (Yin and Wunderink, 2018). HCoVs contain single-stranded, polycistronic RNA genomes of positive polarity (∼30 kb). These viral genomes are translated into multiple nonstructural proteins (ORF1a and ORF1b), structural proteins (S, E, M, and N), and lineage-specific accessory proteins showing differences in these viruses. For instance, in the case of SARS-CoV, accessory proteins include ORF3a, ORF3b, ORF6, ORF7a, ORF7b, ORF8a, ORF8b, and ORF9b (Fung et al., 2020).

The most common clinical symptoms in SARS-CoV-2 include fever, cough, dyspnea, fatigue, headache, myalgia, and diarrhea. Some patients afterward suffer from shortness of breath and recurrent or ongoing fever. In nearly 13% of patients, intense care treatment (e.g., mechanical ventilation) should be applied (Lai et al., 2020; Wang D. et al., 2020). The pathobiology of SARS-CoV-2 and related molecular mechanisms behind the coronavirus-associated lung injury are not yet completely understood; however, the role of some key molecular intermediates are not deniable (Marini and Gattinoni, 2020). Among those signaling mediators, TNF-α, IL-1, IL-6, IL-8, and IL-1β, NF-κB, MMPs, MAPK, and COX-2 seem to play critical roles in the pathogenesis of COVID-19 and associated lung injury (Fakhri et al., 2020b; Liu Y. et al., 2020; Merad and Martin, 2020). In terms of ROS, iNOS, as well as nuclear factor erythroid 2-like 2 (Nrf2), autophagy-related molecules (LC-3II, Atg5, and Beclin1), and Janus kinase-signal transducers and activator of transcription (JAK/STATs) pathway have shown an important role (Seif et al., 2017). From the other point of view, the extracellular signal-regulated kinase (ERK) and protein kinase B (Akt) signaling pathways are of the other dysregulated mediators following lung injury (Mo et al., 2014; Tsai et al., 2015; Jin et al., 2018). In COVID-19 patients, angiotensin-converting enzyme 2 (ACE2) receptor, located on alveolar epithelial cells, has attracted growing attention, as a high-affinity receptor and cotransporter for SARS-CoV-2 entrance to the lung (South et al., 2020; Ziai et al., 2020). Dysregulation of ACE2/Ag (1–7)/Mas receptor and ACE1/Ag II/Ag II type 1 receptor pathways could enhance ACE2, thereby increasing the chances of the viral entry (Rico-Mesa et al., 2020; South et al., 2020). Besides, the dysregulation of ACE2 by SAR-CoV-2 infection inhibits the degradation of Ag II into angiotensin (Ag) (1–7), exacerbates inflammation, and leads to vascular permeability, as well as cardiovascular/lung complications (Leung et al., 2020; South et al., 2020). Based on the pathological findings, an edematous lung with increased weight was also observed in this disease (Ding et al., 2003; Nicholls et al., 2003). Large multinucleated cells (macrophages and pneumocytes) and atypical enlarged pneumocytes comprise large nuclei, prominent nucleoli, and amphophilic granular cytoplasm, which have often been observed in the lungs of SARS patients. However, none of these signs can be considered as a unique feature of SARS-related pathology. The other pathological features usually observed in SARS include squamous metaplasia of bronchial and alveolar epithelial cells; cilia loss of bronchiolar epithelial cells; subpleural multiplication of fibrogranulative tissue in small airways and airspaces; vascular injury hemophagocytosis in residing mononuclear cells in pulmonary tissue; and apoptosis in epithelial cells, lymphocytes, monocytes/macrophages, and pneumocytes (Gu and Korteweg, 2007). Apart from a respiratory infection, gastrointestinal and central nervous system (CNS) infection was also reported in some patients suffering from SARS (Fung et al., 2020). Additionally, in most SARS autopsies, both extensive necrosis of the spleen and atrophy of the white pulp were reported. Reduction of CD4^+^, CD8^+^, and CD20^+^ lymphocytes, dendritic cells, macrophages, and natural killer cells residing in the spleen, as well as atrophy and decrement of the lymph nodes lymphocytes, were often observed. The presence of SARS-CoV was also confirmed in circulating monocytes and T lymphocytes and to some degree in B lymphocytes and natural killer cells (Chong et al., 2004; Gu et al., 2005). The liver is another organ that is affected during the course of this disease. For example, the increment of serum alanine aminotransferase level in SARS patients was associated with some adverse outcomes. Besides, hemophagocytosis or bone marrow hypoplasia, destruction of epithelial cells in the thyroid glands, myofiber necrosis and atrophy of skeletal muscle tissue, and necrosis and vacuities of the adrenal medulla can occur in some SARS patients (Gu and Korteweg, 2007).

## Phytochemicals Against Coronaviruses

**FIGURE 1:**
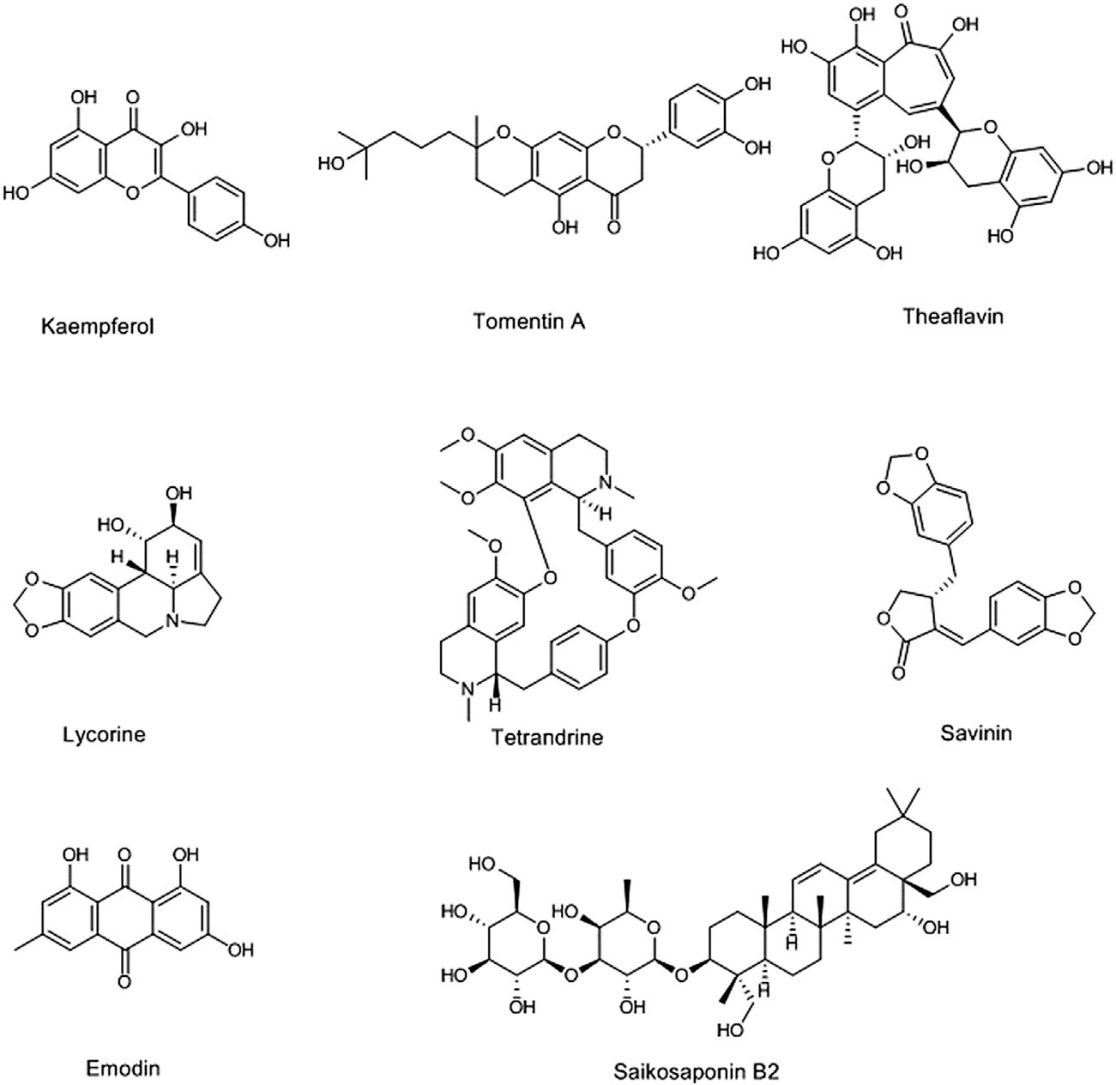
Selected chemical structures of some phytochemicals with potential anti-CoV effects.

Since the outbreak of COVID-19 happened, several researchers have focused on the use of natural compounds for the treatment of related complications. Most of those studies are *in vitro* and *in vivo* screening of phytochemicals against coronaviruses (especially SARS-CoV-2), computer docking models studies on predicting the anti-CoVs effects of these compounds against the coronavirus family members such as SARS-CoV, MERS-CoV, and SARS-CoV-2 (Mani et al., 2020; Zhang D.-h. et al., 2020). According to those studies, natural polyphenol compounds such as quercetin (Chiow et al., 2016), kaempferol (Schwarz et al., 2014), myricetin (Yu et al., 2012), apigenin (Ryu et al., 2010a), and resveratrol (Wahedi et al., 2020) have prominent activities against coronaviruses. Cho and coworkers showed that the geranylated flavonoids (tomentin A-E) isolated from *Paulownia tomentosa* (Thunb.) Steud. (Paulowniaceae) inhibited the papain-like protease as a vital enzyme for SARS-CoV propagation (Cho et al., 2013). In addition, three flavonoid compounds including apigenin-7-O-rhamnoglucoside, herbacetin, and pectolinarin at the concentration of 20 µM blocked the crucial enzyme for SARS-CoV replication, 3C-like protease (Jo et al., 2020). Also, 3C-like protease was inhibited with ten polyphenols isolated from *Broussonetia papyrifera* (L.) L’Hér. ex Vent. (Moraceae), especially with papyriflavonol A at 3.7 µM (Park et al., 2017). On the other hand, the molecular docking study on traditional Chinese medicinal compounds against SARS-CoV-2 showed that the theaflavin, as a flavonoid compound isolated from black tea, *Camellia sinensis* (L.) Kuntze (Theaceae), via the inhibition of the SARS-CoV-2 RNA-dependent RNA polymerase, can exert anticoronavirus activities (Lung et al., 2020). Also, hesperidin, which is abundant in citrus, has shown a potential inhibitory effect on ACE2; thereby it could be a good candidate for clinical trials on SARS-CoV-2 (Haggag et al., 2020). Besides, alkaloids have also shown antiviral effects against coronaviruses. Lycorine, as an indolizidine alkaloid isolated from *Lycoris radiata* (L’Hér.) Herb. (Amaryllidaceae), showed anti-SARS-CoV activities at 15.7 nM (Li et al., 2005). Gyebi and coworkers introduced the 10-hydroxyusambarensine, an indole alkaloid isolated from *Strychnos usambarensis* Gilg ex Engl. (Loganiaceae), and 6-oxoisoiguesterin, a bisnorterpenes isolated from *Salacia madagascariensis* (Celastraceae) as the anti-SARS-CoV-2 agents, through their highest affinity in binding to 3C-like protease obtained from a docking study (Gyebi et al., 2020). Tetrandrine (0.33 µM), fangchinoline (1.01 µM), and cepharanthine (0.83 µM), bisbenzylisoquinoline alkaloids of *Stephania tetrandra* S. Moore (Menispermaceae), are other phytochemicals that showed the anti-HCoV activities (Kim et al., 2019). Sanguinarine, palmatine, berberine, chelidonine, jatrorrhizine, ipecac alkaloids, and emetine are other alkaloids that are suggested as anti-SARS-CoV-2 agents (Bleasel and Peterson, 2020; Wink, 2020). Besides, among other anti-CoVs phytochemicals saikosaponins (A, B2, C, D) as triterpene saponin glycosides of Bupleurum spp. (Apiaceae) have shown hopeful results. The saikosaponin B_2_ at 6 μM, in addition to possessing an anti-CoV effect, also showed inhibitory effects on the virus propagation stages (Cheng et al., 2006). Glycyrrhizin is another triterpene saponin, obtained from *Glycyrrhiza glabra* L. (Fabaceae), with anti-CoV activity and blocking effects on several steps of viral replication such as permeation and adsorption (Bailly and Vergoten, 2020; Cinatl et al., 2003). Triterpenoids of *Euphorbia neriifolia* L. (Euphorbiaceae) also indicated anti-HCoV effects. Among these triterpenoids, friedelane derivatives showed the greatest effect (Chang et al., 2012). The phytochemicals in essential oils are among the other anti-HCoV natural compounds (Nadjib, 2020). Jensenone and 1,8-cineole, as monoterpenes in *Eucalyptus* spp. (Myrtaceae) essential oil, showed anti-CoV effects in docking studies (Sharma and Kaur, 2020a; Sharma and Kaur, 2020b). Savinin, a lignoid isolated from *Chamaecyparis obtusa* (Siebold & Zucc.) Endl. (Cupressaceae), and betulinic acid, a triterpenoid of *Betula spp.* (Betulaceae), showed anti-SARS-CoV activities via the inhibition of 3CL protease at 25 and 10 μM, respectively (Wen et al., 2007). Also, quinones such as emodin, aloe-emodin, and *Tripteryguim regelii* (Celastraceae) quinones including celastrol, pristimererin, tingenone, and iguesterin showed the anti-SARS-CoV activities. The emodin, aloe-emodin, and iguesterin inhibited the 3CL protease at 20, 366, and 2.6 µM, respectively (Lin et al., 2005; Ryu et al., 2010b; Schwarz et al., 2011). In addition, emodin, isolated from *Rheum officinale* Baill. and *Polygonum multiflorum* (Thunb.) Moldenke (Polygonaceae), inhibited interaction between ACE2 and S protein at 200 µM. With mention to the above, it can be said that phytochemicals are potential sources for the discovery of effective drugs against coronaviruses, especially anti-SARS-CoV-2. For this purpose, several clinical trials on phytochemicals such as polyphenols (NCT04400890), hesperidin and diosmin (NCT04452799), resveratrol (NCT04542993, NCT04536090, and NCT04377789), quercetin (NCT04468139 and NCT04377789), artemisinin and curcumin (NCT04382040), epigallocatechin gallate (NCT04446065), glycyrrhizin (NCT04487964), colchicine (NCT04527562, NCT04392141, NCT04375202, NCT04355143, and NCT04360980), berberine (NCT04479202), and tetrandrine (NCT04308317) have been designed and are going on. The structures of some anti-CoV phytochemicals are shown in Figure 1.

## Phytochemicals as Potential Agents for Coronavirus-Associated Lung Injury

Medicinal plants and isolated phytochemicals can cover multiple therapeutic targets at the same time and lie in the fact that they are widely used in the treatment of various diseases, including viral diseases and related complications. Since infection with any of the viruses of the Coronaviridae family, including SARS-CoV-2, can cause severe damage to the pulmonary system (Ding et al., 2003), the plant-derived secondary metabolites can play a significant role in reducing these pulmonary complications. The phytochemicals with different molecular targets and signaling mechanisms, including reducing proinflammatory and oxidant mediators such as TNF-α, IL-1, IL-6, IL-8, IL-1β, NF-κB, MMPs, iNOS, MAPK, COX-2, and ROS, minimize lung injury. Therefore, protective effects on lung injury, along with other effects, including antiviral (especially anti-CoVs) effects, have attracted the attention of many researchers on the use of phytochemicals as potential strategies for discovering new anti-CoV agents regarding controlling related complication (Bellik et al., 2012; Cornélio Favarin et al., 2013b).

### Alkaloids

**FIGURE 2 F2:**
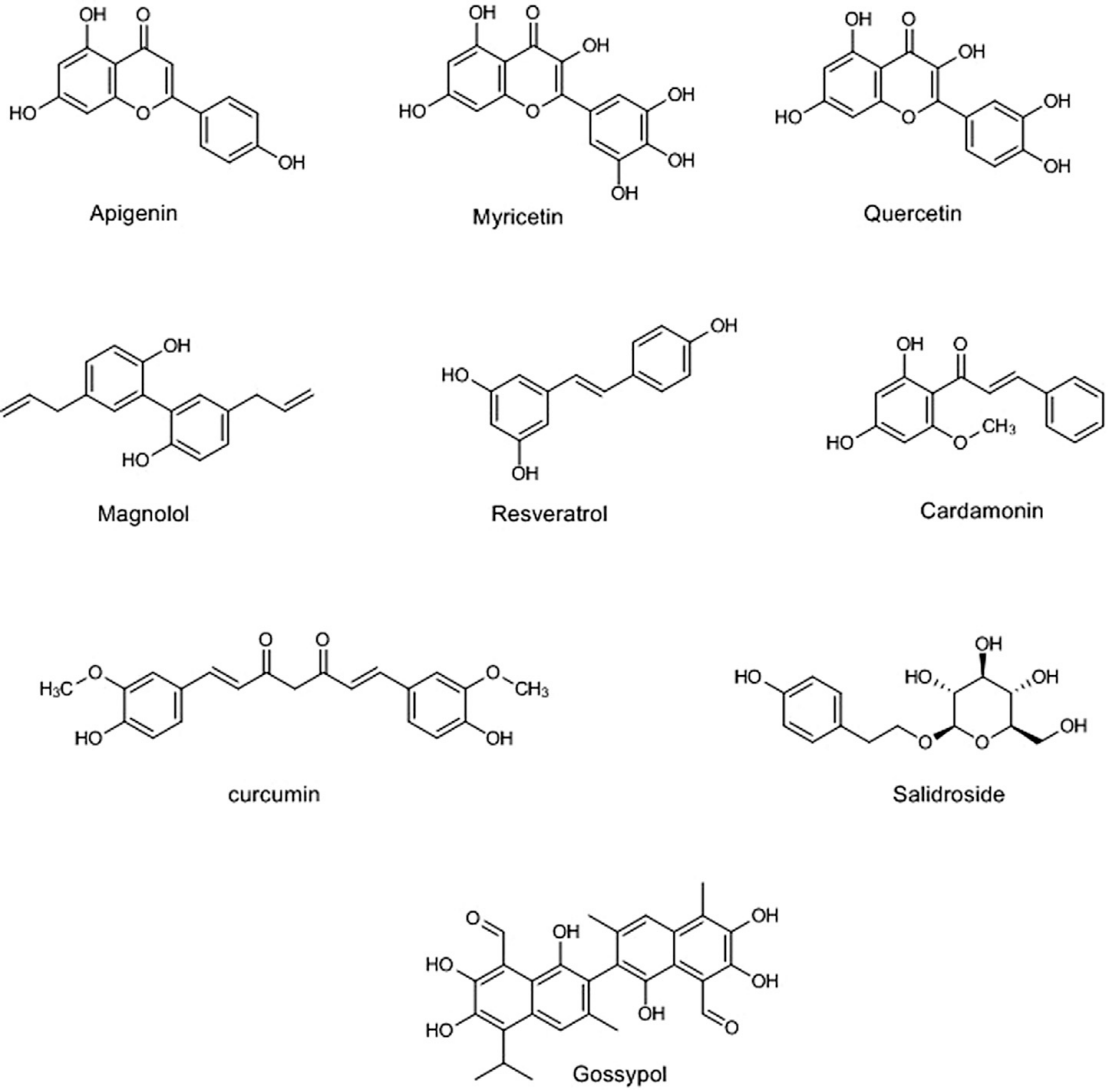
Selected chemical structures of polyphenols with potential protective effects against lung injury.

Alkaloids are one of the largest classes of natural products that are mainly found in several plant families such as Solanaceae, Ranunculaceae, Rubiaceae, Papaveraceae, Amaryllidaceae, and Fabaceae. The main feature of this group is the presence of the nitrogen atom in their structure (Yang and Stöckigt, 2010). Several studies showed that alkaloids have the potential of reducing lung injury induced by different methods. Sinomenine (Figure 2) is an isoquinoline alkaloid that is isolated from the stem and rhizome of *Sinomenium acutum* (Thunb.) Rehder & E.H.Wilson (Menispermaceae). It reduced the lung injury induced by lipopolysaccharides (LPS) and *Escherichia coli*, via regulation of inflammatory signaling pathway, including the downregulation of IL-6, IL-1β, TNF-α, NF-κB, iNOS, and COX-2 and upregulation of the protective anti-inflammatory adenosine A2A receptor. Sinomenine also inhibited oxidative stress markers, including the increase of the superoxide dismutase (SOD) and the decrease of the malondialdehyde (MDA) (Li et al., 2013; Liu S. et al., 2018). Besides, sinomenine [100 mg/kg, intraperitoneally (i.p.)] upregulated the expression of Nrf2 and autophagy-related molecules (LC-3II, Atg5, and Beclin1), as critical mediators in increasing cell resistance against oxidative stress and inflammation, 1 h after inducing lung injury by LPS (8 mg/kg) in mice. Moreover, lung wet/dry (W/D) ratio, pulmonary edema, and the protein leakage into bronchoalveolar lavage fluid (BALF), as the pathological markers of lung injury, were decreased by sinomenine (Wang X. et al., 2019). In addition, six isosteroid alkaloids (imperialine, verticinone, verticine, imperialine-3-β-D-glucoside, delavine, and peimisine) and total alkaloid extraction isolated from bulbs of *Fritillaria cirrhosa* D.Don (Liliaceae) showed the protective effects on lung injury, induced by LPS and cigarette smoke, increase the expression of Nrf2 and heme oxygenase (HO-1), and reduce ROS production, IL-6, and TNF-α expression *in vivo* and *in vitro* (Wang et al., 2016; Liu S. et al., 2020).

Toll-like receptor 4 (TLR4) is an inflammatory signaling pathway whose expression is increased in acute lung injury (Yang H.-Z. et al., 2012). Sophocarpine (25 and 50 mg/kg, i.p.), quinolizidine alkaloid isolated from the seeds of *Sophora alopecuroides* L. (Fabaceae), reduced LPS-induced lung injury in mice by the inhibition of TLR4 expression (Lu et al., 2019).

Zhang et al. reported that tabersonine, as a monoterpenoid indole alkaloid isolated from the root of *Catharanthus roseus* (L.) G.Don (Apocynaceae), has shown a protective effect on lung injury induced by LPS *in vivo* (20 mg/kg, i.p.) and *in vitro* (mouse bone marrow-derived macrophages, 10 µM). This study showed that tabersonine decreased the expression of TNF receptor-associated factor 6 (TRAF6) and thereby blocked p38MAPK-activated protein kinase 2 (MAPK/MK2) and NF-κB activities. The amelioration of the aforementioned signaling pathways/mediators leads to the inhibition of proinflammatory mediators and the reduction of pathological indices of lung injury such as total protein concentrations in BALF ameliorated lung injury (Zhang et al., 2018).

Berberine, an isoquinoline alkaloid isolated from different species such as *Berberis vulgaris* L. (Berberidaceae) and *Coptis chinensis* Franch. (Ranunculaceae), has indicated protective effects on LPS-induced lung injury via activating Nrf2 and increasing the expression of HO-1 in C57BL/6 mice at 10 mg/kg (i.p., 24 and 2 h before injection of LPS, 2.5 mg/kg), as well as the *in vitro* manner on the human bronchial epithelial cell line at 5 and 10 μM concentrations. Berberine also reduced the pulmonary edema and the protein leakage into BALF of mice (Liang et al., 2019).

Matrine (tetracycloquinolizindine) (Li W. W. et al., 2019), antidesmone (tetrahydroquinoline) (Lu et al., 2017), cepharanthine (bisbenzylisoquinoline) (Huang et al., 2014), epigoitrin (pyrrolidine) (Luo et al., 2019), isotetrandrine (bisbenzyltetrahydroisoquinoline) (Liang et al., 2014), neferine (bisbenzylisoquinline) (Zhao et al., 2010), and oxysophoridine (quinolizidine) (Fu et al., 2015) are other alkaloids that have shown anti-lung injury effects evaluated by *in vivo* and *in vitro* experiments. Accordingly, they regulated the proinflammation mediators and oxidative markers (Table 1). [Fig F2] [Fig F4] show the chemical structures and schematic diagram of the possible mechanisms of action of some alkaloids and other phytochemicals with protective effects against lung injury, respectively. Generally, the alkaloids especially quinolines and quinazolines have shown therapeutic effects on lung injury via inhibiting the MAPKs pathway and their interconnected mediators, including TLR4, and inflammatory cytokines such as IL-1β, IL-6, and TNF-α. These agents have also been shown to enhance the Nrf2/HO-1 pathway, glutathione, and SOD as antioxidative stress markers. Therefore, this impressive role on lung injury, along with the other beneficial roles of the alkaloids especially their antiviral effects, introduces these compounds as the multitarget agents for the treatment of coronavirus infection and their complications.

**TABLE 1:** Phytochemicals and their mechanisms of action against lung injury.

Phytochemical class	Compounds	Natural source	Mechanisms	Type of study	Lung injury model	Antiviral activity	References
Alkaloid	Antidesmone	*Antidesma membranaceum* Müll.Arg. (Euphorbiaceae)	↓TNF-α, ↓IL-6, ↓IL-1β, ↓NF-κB, ↓MAPK, ↓COX-2, ↓iNOS, ↓wet/dry ratio of lungs, ↓JNK, ↓ p38	*In vitro*/*In vivo*	LPS	NR	Lu et al. (2017)
Alkaloid	Berberine	*Berberis vulgaris* L. (Berberidaceae)	↑Nrf2, ↑HO-1, ↓MPO, ↓TGF-β1, ↓ROS, ↓wet/dry ratio of lungs	*In vitro*/*In vivo*	LPS, radiation	Yes	Yan et al. (2018), Liang et al. (2019)
Alkaloid	Cepharanthine	*Stephania cepharantha* Hayata (Menispermaceae)	↓TNF-a, ↓IL-6, ↓IL-1β, ↓NF-κB, ↓IκBα, ↓ERK, ↓MAPK,↓ MPO	*In vitro*/*In vivo*	LPS	Yes	Zhang et al. (2005), Huang et al. (2014)
Alkaloid	Epigoitrin	*Isatis tinctoria L.* (Brassicaceae)	↓Viral replications, ↓MFN2, ↑MAVS, ↑IFN-b, ↑IFITM3, ↓TNF-a, ↓IL-1β	*In vitro*/*In vivo*	Influenza virus	Yes	Luo et al. (2019)
Alkaloid	Isotetrandrine	*Fritillaria cirrhosa* D.Don (Liliaceae)	↓TNF-a, ↓IL-6, ↓IL-1β, ↓NF-κB, ↓NF-κB. ↓MAPK, ↓MPO, ↓wet/dry ratio of lungs	*In vitro*/*In vivo*	LPS	NR	Liang et al. (2014)
Alkaloid	Matrine	*Sophora flavescens* Aiton (Fabaceae)	↓TNF-a, ↓IL-6, ↓HMGB1, ↓MPO, ↓wet/dry ratio of lungs, ↓MDA, ↓ROS, ↓NF-κB	*In vitro*/*In vivo*	LPS	Yes	Yang Y. et al. (2012), Li W. W. et al. (2019)
Alkaloid	Neferine	*Nelumbo mucifera* Gaertn. (Nelumbonaceae)	↑SOD,↑MDA, ↓MPO, ↓TNF-a, ↓IL-6, ↓NF-κB, ↓TGF-b1	*In vitro*/*In vivo*	Bleomycin	NR	Zhao et al. (2010)
Alkaloid	Oxysophoridine	*Siphocampylus verticillatus* (Cham.) G.Don (Campanulaceae)	↓TNF-α, ↓IL-6, ↓IL1β, ↓wet/dry ratio of lungs, ↓NF-κB, ↓pulmonary cell apoptosis	*In vivo*	LPS	Yes	Fu et al. (2015), Zhang Y.-N. et al. (2020)
Alkaloid	Sinomenine	*Sinomenium acutum* (Thunb.) Rehder & E.H.Wilson (Menispermaceae)	↓IL-6, ↓IL-1β, ↓TNF-α, ↓NF-κB, ↓iNOS, ↓COX-2, ↑SOD, ↓MDA, ↑Nrf2, ↑ LC-3II, ↑Beclin1, ↓lung wet/dry ratio, ↓pulmonary edema, ↓BALF	*In vivo*	LPS, sepsis	NR	Li et al. (2013), Liu S. et al. (2018), Wang W. et al. (2020)
Alkaloid	Tabersonine	*Catharanthus roseus* (L.) G.Don (Apocynaceae)	↓TRAF6, ↓MAPK/MK2, ↓NF-κB, ↓TNF-α, ↓IL-6, ↓IL-1β, ↓MPO, ↓iNOS, ↓NO	*In vitro*/*In vivo*	LPS	NR	Zhang et al. (2018)
Anthocyanin	Cyanidin	*Vaccinium corymbosum* L. (Ericaceae)	↓TNF-α, ↓IL-6,↓IL-1β, ↓NF-κB, ↓COX-2, ↓PGE2	*In vivo*	Sepsis	Yes	Liu et al. (2015), Yan et al. (2015), Pour et al. (2019)
Anthocyanin	Malvidin	*Vaccinium corymbosum* L. (Ericaceae)	↓Bax/Bcl-2, ↓Caspase-3, ↓IL-1β, ↓TNF-α	*In vivo*	Radiation	Yes	Liu et al. (2015), Yan et al. (2015), Pour et al. (2019)
Carbohydrate	Polysaccharides	*Houttuynia cordata* Thunb. (Saururaceae)	↓TNF-α, ↓wet/dry ratio of lungs, ↓TLR4,.↓TNF-α, ↓IL-6, ↓IL-1β, ↓MPO	*In vivo*	LPS	NR	Cheng et al. (2012), Xu et al. (2015)
Chalcone	Cardamonin	*Alpinia katsumadai* K.Schum. (Zingiberaceae)	↓TNF-α, ↓IL-6, ↓IL-1β, ↓P38 MAPK, ↓MPO, ↓wet/dry ratio of lungs	*In vitro*/*In vivo*	Sepsis	NR	Wei et al. (2012)
Coumarin	Anomalin	*Saposhnikovia divericata* (Turcz. ex Ledeb.) Schischk (Apiaceae)	↓IL-1β, ↓IL-6, ↓TNF-α, ↑GST, ↑GSH, ↑catalase, ↓MDA, ↓NO, ↓wet/dry ratio of lungs	*In vitro*/*In vivo*	LPS	NR	Khan et al. (2019)
Coumarin	Daphnetin	*Daphne* spp. (Thymelaeaceae)	↓NF-κB, ↓TNF-α, ↓IL-6, ↓IL-1β, ↓JAK/STATs, ↓ROS, ↓MPO, ↓MAPK	*In vitro*/*In vivo*	LPS	NR	Yu et al. (2014), Seif et al. (2017), Shen et al. (2017)
Coumarin	Esculetin	*Artemisia capillaris* Thunb. (Asteraceae)	↓IL-23, ↓TNF-α, ↓IL-6, ↓IL-1β, ↓MAPK, ↓neutrophils, ↓NF-κB, ↓macrophages, ↓ERK, ↓Akt	*In vivo*	LPS	Yes	Galabov et al. (1996), Lee et al. (2020)
Coumarin	Fraxin	*Fraxinus chinensis* subsp. *Rhynchophylla* (Hance) A.E.Murray (syn. *Fraxinus rhynchophylla* Hance) (Oleaceae)	↓NLRP3, ↓wet/dry ratio of lungs, ↓NF-κB, ↓MPO, ↓MDA, ↓SOD, ↓IL-1β, ↓ IL-6, ↓TNF-α	*In vivo*	LPS	NR	Li W. et al. (2019)
Coumarin	Isofraxidin	*Sarcandra glabra* (Thunb.) Nakai (Chloranthaceae)	↓PGE2, ↓COX-2, ↓NF-κB, ↓IL-1β, ↓IL-6, ↓MIP-2, ↓wet/dry ratio of lungs, ↓MPO, ↓MAPK, ↓AKT	*In vitro*/*In vivo*	LPS, influenza virus	Yes	Jin et al. (2020), Majnooni et al. (2020)
Coumarin	Osthole	*Cnidium monnieri* (L.) Cusson (Apiaceae)	↓IL-1β, ↓IL-6, ↓TNF-α, ↓NF-κB, ↓ERK, ↓Akt, ↓wet/dry ratio of lungs	*In vitro*/*In vivo*	LPS, neutrophil oxidative stress, hemorrhagic shock, intestinal ischemia reperfusion	Yes	Shi et al. (2013), Shokoohinia et al. (2014)
Coumarin	Praeruptorin D and E	*Peucedanum praeruptorum *Dunn (Apiaceae)	↓NF-κB, ↓IL-6, ↓TNF-α, ↓neutrophils, ↓cells infiltration in BALF, ↓MPO	*In vivo*	LPS, hydrochloric acid	NR	Yu et al. (2013)
Coumarin	Psoralidin	*Cullen corylifolium* (L.) medik. (syn. *Psoralea corylifolia* L.) (Fabaceae)	↓COX-2, ↓5-LOX, ↓IL-1β, ↓IL-6, ↓TNF-α, ↓TGF-b	*In vitro*	Ionizing radiation	Yes	Yang H. J. et al., (2011), Kim et al., (2014)
Coumarin	Scoparone	*Artemisia capillaris* Thunb. (Asteraceae)	↓wet/dry ratio of lungs, ↓TLR4, ↓NF-κB, ↓IL-1β, ↓IL-6, ↓TNF-α, ↓MPO	*In vitro*/*In vivo*	LPS	NR	Niu et al. (2014)
Coumarin	Umbelliferone	*Petroselinum crispum* (Mill.) Fuss (Apiaceae)	↓MCP-1, ↓MPO, ↓MDA, ↑SOD, ↓TLR4, ↓MyD88, ↓NF-κB, ↓wet/dry ratio of lungs	*In vivo*	LPS	NR	Wang D. et al. (2019)
Flavonoid	Apigenin	*Citrus × aurantium* L. [syn. *Citrus sinensis* (L.) Osbeck] (Rutaceae)	↓TNF-α, ↓wet/dry ratio of lungs, ↓IL-6, ↓IL-1β, ↓NF-κB, ↓TLR4, ↓ MPO	*In vivo*	LPS	Yes	Shibata et al. (2014), Li et al. (2018)
Flavonoid	Breviscapine	*Erigeron breviscapus* (Vaniot) Hand.-Mazz (Asteraceae)	↓ICAM-1, ↓IL-18	*In vivo*	Left heart ischemic reperfusion	NR	Wang et al. (2013)
Flavonoid	Daidzein	*Glycine max* (L.) Merr (Fabaceae)	↓TLR4, ↓MyD88, ↓NF-κB, ↓MPO, ↓wet/dry ratio of lungs, ↓IL-6, ↓TNF-α	*In vitro*/*In vivo*	LPS	Yes	Feng et al. (2015), Seo et al. (2016)
Flavonoid	Eriodictyol	*Dracocephalum rupestre* Hance (Lamiaceae)	↑Nrf2, ↓MPO, ↓TNF-α, ↓IL-6, ↓IL-1β, ↓MIP-2, ↓wet/dry ratio of lungs	*In vivo*	LPS	NR	Zhu et al. (2015)
Flavonoid	Fisetin	*Fragaria x ananassa* (Duchesne ex Weston) Duchesne ex Rozier (Rosaceae)	↓Neutrophils, ↓macrophages, ↓TNF-α, ↓IL-6, ↓IL-1β, ↑Nrf2, ↑GPx, ↑SOD, ↑CAT	*In vivo*	Cigarette smoke	Yes	Lin et al. (2012), Hussain et al. (2019)
Flavonoid	Hesperetin	*Citrus × aurantium* L. [syn. *Citrus sinensis* (L.) Osbeck] (Rutaceae)	↓TNF-α, ↓IL-6, ↓MPO, ↓LDH, ↑SOD, ↓TLR4, ↓MyD88, ↓NF-κB	*In vivo*	LPS	NR	Wang X. et al. (2019)
Flavonoid	Hyperin	*Abelmoschus manihot* (L.) medik (Malvaceae)	↓inflammatory cell infiltration, ↓MPO activity, ↓TNF-α, ↓IL-6, ↓ IL-1β, ↓NF-κB, ↓wet/dry ratio of lungs	*In vivo*	LPS	Yes	Wu et al. (2007), Hu et al. (2019)
Flavonoid	Isorhamnetin	*Hippophae rhamnoides* L. (Elaeagnaceae)	↓Pulmonary edema, ↓TNF-α, ↓IL-6, ↓IL-1β, ↓ERK, ↓JNK, ↓NF-κB	*In vitro*/*In vivo*	LPS	Yes	Dayem et al. (2015), Chi et al. (2016)
Flavonoid	Kaempferol	*Malus domestica* (Suckow) Borkh*.* (Rosaceae)	↓Pulmonary edema, ↓TNF-α, ↓IL-6, ↓IL-1β, ↓alveolar wall thickness, ↓alveolar ↓hemorrhage, ↓leukocytes infiltration, ↑SOD,↓NF-κB,↓MAPKs, ↓MPO, ↓wet/dry ratio of lungs	*In vivo*	LPS	Yes	Guan et al. (2012), Schwarz et al. (2014)
Flavonoid	Luteolin	*Lonicera japonica* Thunb. (Caprifoliaceae)	↓Neutrophil chemotaxis, ↓MPO, ↓respiratory, ↓Akt, ↓ERK, ↑Nrf2, ↓NF-κB, ↑GPx, ↑SOD, ↑CAT	*In vivo*	Mercuric chloride, LPS	Yes	Lee et al. (2010), Liu B. et al. (2018), Yan et al. (2019)
Flavonoid	Myricetin	*Solanum lycopersicum* L. (Solanaceae)	↓TLR4, ↓MyD88, ↓NF-κB, ↓MPO,↓inflammatory cell migration, ↑SOD, ↑GPx, ↑CAT, ↓MPO, ↓wet/dry ratio of lungs, ↓IL-6, ↓TNF-α	*In vivo*	LPS	Yes	Ong and Khoo (1997), Mao and Huang (2017)
Flavonoid	Naringenin	*Citrus × aurantium L.* (syn. *Citrus paradisi* Macfad.) (Rutaceae)	↓PI3K, ↓Akt, ↓MAPK, ↓pulmonary edema, ↓ROS, ↓TNF-α, ↓MPO, ↓IL-6, ↓IL-1β	*In vitro*/*In vivo*	LPS, acid	Yes	Lee et al. (1999), Mao and Huang (2017), Zhao et al. (2017), Yu et al. (2020)
Flavonoid	Quercetin	*Myrsine melanophloeos* (L.) R.Br. ex Sweet (syn. *Rapanea melanophloeos* (L.) Mez) (Primulaceae)	↓NF-κB, ↓JNK/SAPK, ↓p38, ↓p44/p42, ↑caspase-3	*In vivo*	Radiation	Yes	Wang J. et al. (2015), Chiow et al. (2016)
Flavonoid	Rutin	*Fagopyrum esculentum* Moench (Polygonaceae)	↓NF-κB, ↓MAPK, ↑GPx, ↑SOD, ↑CAT, ↓MIP, ↓MMP-9, ↓Akt	*In vivo*	LPS	Yes	Lin et al. (2012), Chen et al. (2014), Yeh et al. (2014)
Flavonoid	Silymarin	*Silybum marianum* (L.) Gaertn (Asteraceae)	↑Nrf2, ↑HO-1, ↑GPx, ↑SOD, ↑CAT, ↓ MPO	*In vitro*/*In vivo*	Paraquat	Yes	Özçelik et al. (2011), Zhao et al. (2015)
Flavonoid	Wogonin	*Scutellaria baicalensis* Georgi (Lamiaceae)	↓NO, ↓TNF-α, ↓IL-6, ↓IL-1β, ↓iNOS, ↓NF-κB,↓ MPO	*In vitro*/*In vivo*	LPS	Yes	Guo et al. (2007), Yao et al. (2014)
Iridoid	Geniposide	*Gardenia jasminoides* J.Ellis (Rubiaceae)	↓NF-κB, ↓MAPKs,↓ TNF-α, ↓IL-6, ↓alveolar hemorrhage, ↓MPO, ↓wet/dry ratio of lungs	*In vivo*	LPS	Yes	Xiaofeng et al. (2012), Zhang et al. (2017b)
Isothiocyanate	Sulforaphane	*Brassica* spp. (Brassicaceae)	↑Nrf2, ↓PGE2, ↓COX-2, ↓MMP-2, ↓NO, TNF-α, ↓IL-6	*In vivo*	LPS	Yes	Qi et al. (2016), Yu et al. (2016)
Phenolic acid	Caffeic acid	*Coffea arabica* L. (Rubiaceae)	↓MDA, ↑SOD, ↑CAT	*In vivo*	Radiation	Yes	Yildiz et al. (2008), Özçelik et al. (2011)
Phenolic acid	Chicoric acid	*Echinacea purpurea* (L.) Moench (Asteraceae)	↑Nrf2, ↑HO-1, ↓wet/dry ratio of lungs, ↓MPO, ↓MAPK,↓ NLRP3, ↑SOD, ↓NF-κB	*In vivo*	LPS	Yes	Lin et al. (1999), Ding et al. (2019)
Phenolic acid	Chlorogenic acid	*Coffea arabica* L. (Rubiaceae)	↓iNOS, ↓NO, ↓leukocytes, ↓MPO	*In vivo*	LPS	Yes	Zhang et al. (2010), Özçelik et al. (2011)
Phenolic acid	Ellagic acid	*Camellia sinensis* (L.) Kuntze (Theaceae)	↓NF-κB, ↓COX-2, ↑IL-10, ↓IL-6, ↓TNF-α, ↓IL-1β, ↓NF-κB	*In vitro*/*In vivo*	LPS, hydrochloric acid	Yes	Cornélio Favarin et al. (2013a), Park et al. (2014), Guan et al. (2017)
Phenolic acid	Rosmarinic acid	*Rosmarinus officinalis* Spenn. (Lamiaceae)	↓ERK/MAPK, ↓IL-6, ↓TNF-α, ↓IL-1β, ↑SOD	*In vivo*	LPS	Yes	Petersen and Simmonds (2003), Chu et al. (2012)
Phenolic compound	Apocynin	*Picrorhiza kurroa* Royle ex Benth. (Plantaginaceae)	↓TNF-α, ↑ SOD, ↓pulmonary vascular permeability, ↓MDA, ↓NADPH	*In vivo*	LPS	NR	Xu et al. (2014)
Phenolic glycoside	Salidroside	*Rhodiola rosea* L. (Crassulaceae)	↓IL-6, ↓TNF-α, ↓IL-1β, ↓wet/dry ratio of, ↓MPO, ↓NF-κB, ↓TGF-β1	*In vivo*	LPS, paraquat	Yes	Wang et al. (2009), Guan et al. (2012), Zhang et al. (2014)
Phenolic terpene	Cannabidiol	*Cannabis sativa* L. (Cannabaceae)	↓MPO, ↓TNF-α, ↓IL-6	*In vivo*	LPS	Yes	Ribeiro et al. (2012), Ribeiro et al. (2015), Lowe et al. (2017)
Polyphenol	Curcumin	*Curcuma longa* L. (Zingiberaceae)	↓NF-κB, ↓PGE2, ↓inflammatory responses, ↓TGF*-*β, ↓TNF-α, ↓IL-6, ↓MMP-9, ↓PGE2	*In vitro*/*In vivo*	Diabetes, bleomycin, LPS	Yes	Sun et al. (2008), Smith et al. (2010), Suresh et al. (2012), Zorofchian Moghadamtousi et al. (2014), Zhang et al. (2015)
Polyphenol	Gossypol	*Gossypium herbaceum* L. (Malvaceae)	↓IL-6, ↓TNF-α, ↓IL-1β, ↓wet/dry ratio of lungs, ↓p46-p54, ↓JNK, ↓p42, ↓ p44 ERK, ↓p38, ↓NF-κB	*In vivo*	LPS	Yes	Liu Z. et al. (2013), Keshmiri-Neghab and Goliaei (2014)
Polyphenol	Magnolol	*Magnolia officinalis* Rehder & E.H.Wilson (Magnoliaceae)	↓IL-6, ↓TNF-α, ↓IL-1β, ↓NF-κB, ↓TLR4, ↓wet/dry ratio of lungs, ↓MPO	*In vivo*	LPS	Yes	Yunhe et al. (2012), Singla (2014)
Polyphenol	Resveratrol	*Vitis* spp. (Vitaceae)	↓MAPK, ↓PI3K, ↓AKT, ↓MyD88, ↓TLR4, ↓Nrf2, ↓HO-1, ↓wet/dry ratio of lungs, ↓NF-κB, ↓IL-1β, ↓IL-6, ↓TNF-α, ↓ROS, ↓iNOS, ↓ SOD, ↓MDA, ↓MIP-2, ↓IL18, ↓MPO	*In vivo*	LPS, hypoxia, sepsis, staphylococcal enterotoxin B, nickel, methamphetamine, bleomycin, chest trauma, cigarette smoke	Yes	Sovak (2001), Kolgazi et al. (2006), Şener et al. (2007), Cao et al. (2011), Rieder et al. (2012), Bao et al. (2013), Mo et al. (2014), Yu et al. (2014), Jiang et al. (2016), Lin et al. (2017), Torun et al. (2017), Liu S. et al. (2018), Wang et al. (2018), Yang et al. (2018), de Oliveira et al. (2019), Zhu et al. (2019), Özdemir et al. (2019), Cao et al. (2020), Wang X. et al. (2020)
Polyphenol	Tannic acid	*Camellia sinensis* (L.) Kuntze (Theaceae)	↓Wet/dry ratio of lungs, ↓MPO, ↓IL-6, ↓ TNF-α, ↓IL-1β, ↓NF-κB, ↓PGE2	*In vivo*	LPS, sepsis	Yes	Orlowski et al. (2014), Zhang et al. (2019)
Quinone	Emodin	*Rheum palmatum* L. (Polygonaceae)	Activating autophagy, ↓TNF-α, ↓IL-1β, ↓MPO, ↓NO	*In vivo*	LPS	Yes	Schwarz et al. (2011), Dong et al. (2019)
Quinone	Shikonin	*Lithospermum erythrorhyzon* Siebold & Zucc. (Boraginaceae)	↓NF-KB, ↓IL-6, ↓ TNF-α, ↓IL-1β, ↓NO, ↓COX 2, ↓ neutrophil infiltration, ↓wet/dry ratio of lungs	*In vivo*	LPS	Yes	Bai et al. (2013), Liang et al. (2013), Zhang et al. (2017a)
Quinone	Tanshinone IIA	*Salvia miltiorrhiza* Bunge (Lamiaceae)	↓NLRP3, ↓wet/dry ratio of lungs, ↓CO_2_ partial pressure, ↑O_2_ partial pressure	*In vivo*	Oleic acid	Yes	Chen T. et al., (2019), Sun et al. (2019)
Quinone	Thymoquinone	*Nigella sativa* L. (Ranunculaceae)	↓Alveolar infiltration, ↓alveolar edema, ↓iNOS	*In vivo*	Chronic toluene	Yes	Kanter (2011), Fröhlich et al. (2017)
Saponin	Dioscin	*Dioscorea* spp. (Dioscoreaceae)	↓TNF-α, ↓IL-6, ↓IL-1β, ↓MPO, ↓NF-κB	*In vivo*	LPS	Yes	Liu C. et al. (2013), Zeng et al. (2018)
Saponin	Ginsenoside Rg1	*Panax ginseng* C.A.Mey. (Araliaceae)	↓Wet/dry ratio of lungs, ↓proteins, ↓M2 macrophage, ↓pulmonary edema, ↓NF-κB, TNF-α, ↓IL-6, ↓IL-1β	*In vivo*	LPS	Yes	Song et al. (2014), Bao et al. (2015)
Saponin	Ginsenoside Rg3	*Panax ginseng* C.A.Mey. (Araliaceae)	↓NF-κB, ↓COX-2, TNF-α, ↓IL-6, ↓IL-1β, ↓wet/dry ratio of lungs	*In vivo*	LPS	Yes	Song et al. (2014), Cheng and Li (2016)
Saponin	Sodium aescinate	*Aesculus hippocastanum* L. (Sapindaceae)	↓Wet/dry ratio of lungs, ↑SOD, ↑MDA,↓MMP2	*In vivo*	Oleic acid	NR	Menegazzi et al. (2008)
Saponin	Soyasaponin	*Glycine* max (L.) Merr (Fabaceae)	↓COX-2, ↓iNOS, ↓TNF-α, ↓IL-6, ↓IL-1β, ↓NO	*In vivo*	LPS	Yes	Hayashi et al. (1997), Lin et al. (2016)
Terpenoid	Andrographolide	*Andrographis paniculata* (Burm.f.) nees (Acanthaceae)	↓IL-1β, ↑GPx, ↑Nrf2, ↑SOD	*In vivo*	Cigarette smoke	Yes	Guan et al. (2013), Gupta et al. (2017)
Terpenoid	Artemisitene	*Artemisia annua* L. (Asteraceae)	↑Nrf2, ↓TGF-β, ↓MCP-1, ↓IL-6	*In vivo*	Bleomycin	NR	Chen et al. (2016)
Terpenoid	Betulinic acid	*Betula* spp. (Betulaceae)	↑CAT, ↑SOD, ↓iNOS, ↓NO	*In vivo*	Sepsis	Yes	Aiken and Chen (2005), Lingaraju et al. (2015)
Terpenoid	Costunolide	*Lactuca sativa* L. (Asteraceae)	↓TNF-α, ↓IL-6, ↓IL-1β, ↓iNOS, ↓MAPKs	*In vivo*	Heat-killed *S. aureus* (HKSA)	Yes	Chen et al. (1995), Chen Y.-t. et al. (2019)
Terpenoid	Eugenol	*Syzygium aromaticum* (L.) Merr. & L.M.Perry (Myrtaceae)	↓Wet/dry ratio, ↑SOD1, ↑CAT, ↑Gpx1, ↑GST, ↓NF-KB, ↓MPO, ↓IL-6,↓ TNF-α	*In vivo*	LPS	Yes	Pramod et al. (2010), Huang et al. (2015)
Terpenoid	Farnesol	*Prunus persica* (L.) Batsch (Rosaceae)	↑Nrf2, ↑ HO-1, ↓MAPKs, ↓TNF-α, ↓IL-6, ↓IL-1β, ↑GSH, ↑H_2_O_2_, ↓LPO	*In vivo*	Cigarette smoke	Yes	Qamar and Sultana (2008), Ryabchenko et al. (2008)
Terpenoid	Geraniol	*Rosa × damascena* Herrm. (Rosaceae)	↓Wet/dry ratio of lungs, ↓MPO, ↓IL-6,↓TNF-α, ↓IL-1β, ↓ iNOS, ↓COX-2, ↓TLR4, ↓NF-κB, ↓Bax/Bcl-2 ratio	*In vitro*/*In vivo*	LPS	NR	Jiang et al. (2017)
Terpenoid	Glycyrrhizin	*Glycyrrhiza glabra* L. (Fabaceae)	↑ICAM-1, ↓TNF-α,↓IL-1β, ↓MPO, ↓LPO, ↓NF-κB	*In vivo*	Carrageenan	Yes	Menegazzi et al. (2008), Ashfaq et al. (2011)
Terpenoid	Isoforskolin	*Plectranthus hadiensis* (Forssk.) Schweinf. ex Sprenger (syn. *Coleus forskohlii* (Willd.) Briq.) (Lamiaceae)	↓IL-6, ↓TNF-α, ↓IL-1β, ↓wet/dry ratio of lungs, ↓MPO, ↑SOD	*In vivo*	LPS	NR	Yang W. et al. (2011)
Terpenoid	Linalool	*Citrus x aurantium* L. (Rutaceae)	↓IL-6, ↓TNF-α, ↓p38, ↓MAPK, ↓ERK, ↓JNK	*In vitro*/*In vivo*	LPS	NR	Huo et al. (2013)
Terpenoid	Oridonin	*Isodon rubescens* (Hemsl.) H.Hara (syn. *Rabdosia rubescens* (Hemsl.) H.Hara) (Lamiaceae)	↓NLRP3, ↓NF-KB, ↑Nrf2, ↑HO-1, ↑SOD, ↑GSH	*In vitro*/*In vivo*	LPS	Yes	Guo et al. (2013), Yang et al. (2019)
Terpenoid	*p*-Cymene	*Protium* spp. (Burseraceae)	↓IL-6, ↓TNF-α, ↓IL-1β, ↓MPO, ↓NF-KB, ↓MAPKs	*In vivo*	LPS	Yes	Xie et al. (2012), Sharifi-Rad et al. (2017)
Terpenoid	Rubriflordilactone A	*Schisandra sphenanthera* Rehder & E.H.Wilson (syn. *Schisandra rubriflora* Rehder & E.H.Wilson (Schisandraceae)	↓MMP9, ↓iNOS, ↓IL-6, ↓wet/dry ratio of lungs	*In vitro*/*In vivo*	LPS	Yes	Cassels and Asencio (2011), Lingaraju et al. (2015)
Terpenoid	Taraxasterol	*Taraxacum officinale* F.H.Wigg. (Asteraceae)	↓MPO, TNF-α, ↓IL-6, ↓IL-1β, ↓p65, ↓p38, ↓ ERK, ↓JNK, ↓NF-κB	*In vivo*	LPS	Yes	Chowdhury et al. (1990), San et al. (2014)
Terpenoid	Thymol	*Thymus vulgaris* L. (Lamiaceae)	↓NF-KB, ↓IL-6, ↓TNF-α, ↓IL-1β, ↑SOD, ↓MDA, ↓MPO	*In vivo*	LPS	Yes	Sharifi-Rad et al. (2017), Wan et al. (2018)
Terpenoid	Triptolide	*Tripterygium wilfordii* Hook.f. (Celastraceae)	↑Nrf2, ↑HO-1, ↓TLR4, ↓NF-KB, ↓IL-6, ↓TNF-α, ↓IL-1β, ↓MAPKs, ↓MPO	*In vivo*	LPS	Yes	Wan and Chen (2014), Wang et al. (2014)
Terpenoid	Zerumbone	*Zingiber zerumbet* (L.) Roscoe ex Sm (Zingiberaceae)	↑Nrf2, ↑HO-1, ↓LPO, ↓MPO, ↓MMP-9, ↑SOD, ↑GPx, ↑CAT	*In vivo*	LPS	Yes	Dai et al. (1997), Leung et al. (2017)

5-LOX, 5-Lipoxygenase; Akt, protein kinase B; BALF, bronchoalveolar lavage fluid; Bcl-2/Bax, B-cell lymphoma protein 2/associated X; CAT, catalase; CO_2_, carbon dioxide; COX-2, cyclooxygenase-2; ERK, extracellular signal-regulated kinase; GPx, glutathione peroxidase; GSH, glutathione; GST, glutathione S-transferase; HMGB1, high-mobility group box 1 protein; HO-1, heme oxygenase-1; ICAM-1, intercellular adhesion molecule 1; IFITM3, interferon-induced transmembrane protein 3; IFN-b, interferon Beta 1; IL, interleukin; iNOS, inducible nitric oxide synthase; IκBα, inhibitor of nuclear factor-kappa B α; JAK/STATs, janus kinase-signal transducers and activator of transcription; JNK, jun N-terminal kinases; JNK/SAPK, JNK/stress-activated protein kinases; LDH, lactate dehydrogenase; LPS, lipopolysaccharides; MAPK, mitogen-activated protein kinase; MAPK/MK2, MAPK/activated protein kinase 2; MAVS, mitochondrial antiviral signaling; MCP-1, monocyte chemoattractant protein-1; MDA, malondialdehyde; MFN2, mitofusin-2; MIP-2, macrophage inflammatory protein 2; MMPs, matrix metalloproteinases; MPO, myeloperoxidase; MyD88, myeloid differentiation factor 88; NADPH, nicotinamide adenine dinucleotide phosphate; NF-κB, nuclear factor-κB; NLRP3, nucleotide-binding oligomerization domain-like receptors pyrin domain-containing protein 3; NR, not reported; Nrf2, nuclear factor erythroid 2- related factor two; O_2_, oxygen; PGE2, prostaglandin E2; PI3K, phosphoinositide 3-kinases; ROS, reactive oxygen species; SOD, superoxide dismutase; TGF-β1, transforming growth factor β1; TLR4, toll-like receptor 4; TNF-α, tumor necrosis factor-α; TRAF6, TNF receptor-associated factor six.

**FIGURE 3:**
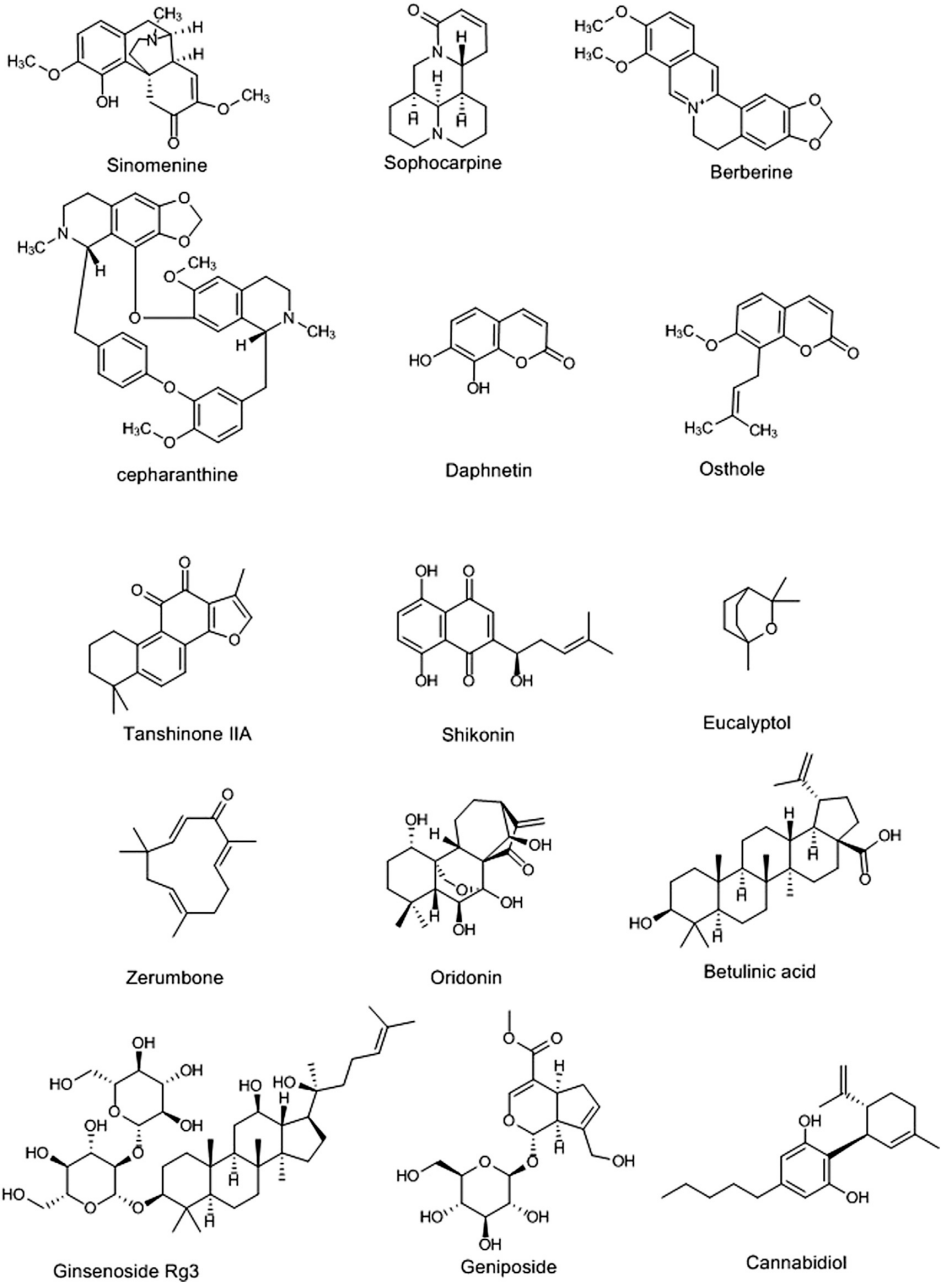
Selected chemical structures of alkaloids, coumarins, terpenes, quinones, and other phytochemicals with potential protective effects against lung injury.

**FIGURE 4 F4:**
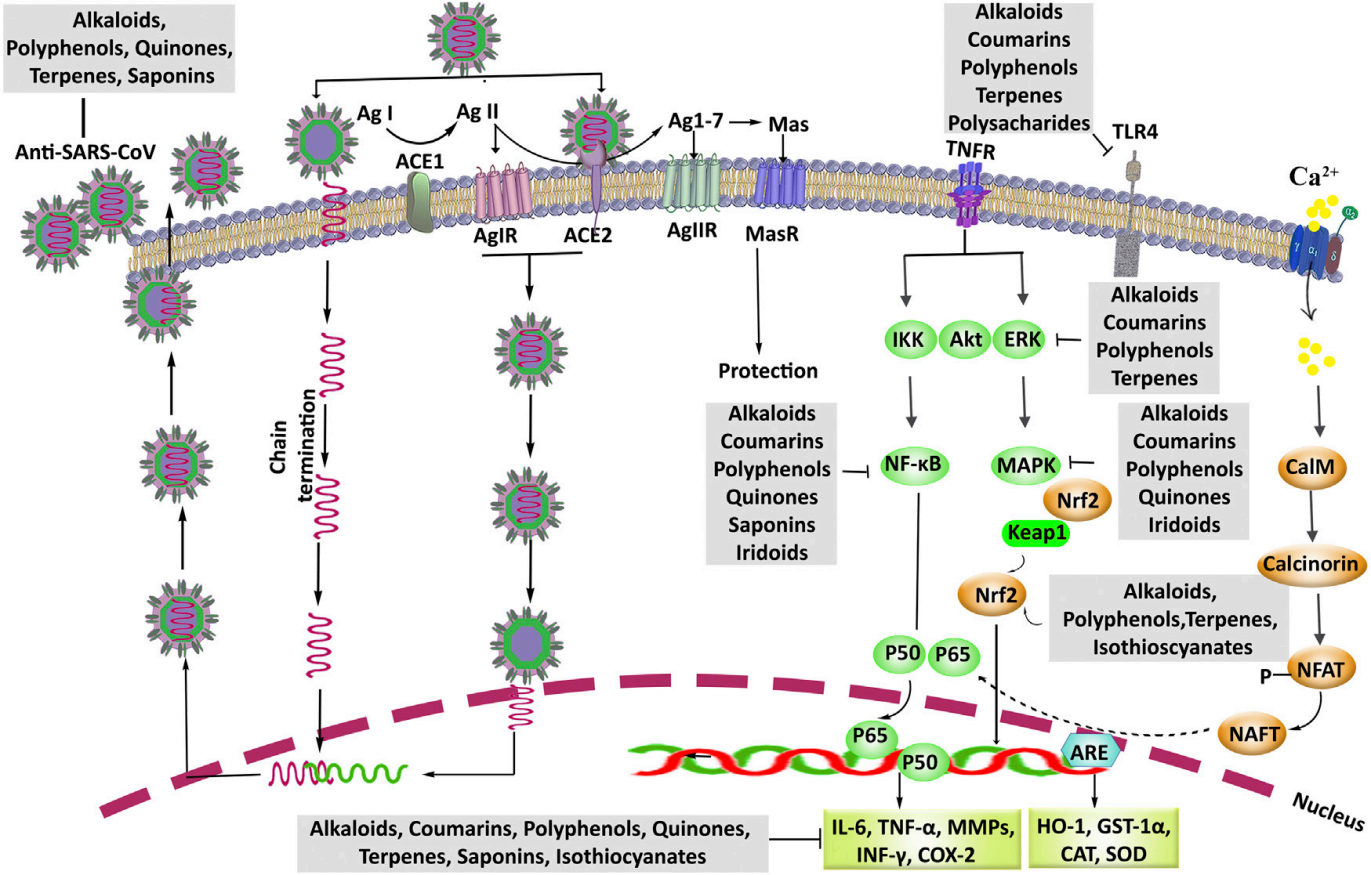
The pharmacological mechanisms and therapeutic targets of phytochemicals against coronavirus-associated lung injury. ACE, angiotensin-converting enzyme; Ag, angiotensin; Akt, protein kinase B; ARE, antioxidant response element; Anti-SARS-CoV, anti-severe acute respiratory syndrome coronavirus; Ca^2+^, calcium; CalM, calmodulin; NAFT, nuclear factor of activated T cells; CAT, catalase; COX-2, cyclooxygenase-2; ERK, extracellular signal-regulated kinase; GSH, glutathione; GST-1α, glutathione S-transferase; HO-1, heme oxygenase-1; IFNγ, interferon γ; IL, interleukin; IKK, inhibitor of nuclear factor-κB (IκB) kinase; MAPK, mitogen-activated protein kinase; MMPs, matrix metalloproteinases; NF-κB, nuclear factor-κB; Nrf2, nuclear factor erythroid 2-related factor 2; SOD, superoxide dismutase; TLR4, toll-like receptor 4; TNF-α, tumor necrosis factor-α; TNFR, TNF receptor.

### Coumarins

Coumarins are the heterocyclic phytochemicals with 2*H*-1-benzopyran-2-one chemical structure. The Apiaceae is one of the greatest plant families that coumarins are isolated from its species (Ribeiro and Kaplan, 2002). Anti-inflammatory and antioxidant properties are two prominent effects of coumarins along with other pharmacological and biological activities such as cytotoxic and anticancer, antiviral, antiangiogenic, anticoagulant, edema-protective, and anxiolytic effect (Fylaktakidou et al., 2004; Venugopala et al., 2013; Srikrishna et al., 2018). The downregulation of inflammatory mediators, including NF-κB, TNF-α, iNOS, and MAPKs pathway, and inhibiting oxidative factors such as ROS and free radicals are critical mechanisms of coumarins. Therefore, coumarinic compounds are thought to exert anti-inflammatory effects on therapeutic applications against lung injury induced by LPS and other destructive inducers (Fylaktakidou et al., 2004; Bansal et al., 2013).

Daphnetin, as a hydroxy coumarin isolated from *Daphne* spp., showed protective effects on lung injury that are induced by LPS *in vivo* and *in vitro*. Daphnetin downregulated the NF-κB pathway via increasing the expression of NF-κB, and TNF-α-induced protein 3, in lung tissues of the C57BL/6 mice (5 mg/kg, i.p.) and the murine peritoneal macrophages (RAW264.7, 160 μM). In another study, pretreatment of mice with 5 mg/kg i.p. of daphnetin significantly reduced protein and cytokines (TNF-α, IL-6, and IL-1β) leakage into BALF that is stimulated with LPS (1 mg/kg). Consequently, daphnetin (80 and 160 μM) suppressed the expression of IL-6 and TNF-α in adenocarcinoma human lung epithelial cell lines (A549), which is induced by LPS (100 ng/ml) (Yu et al., 2014). Also, the regulation of JAK/STATs pathway has a critical role in the production of proinflammatory mediators such as TNF-α, iNOS, COX-2, IL6, and IL-1β (Seif et al., 2017). Daphnetin inhibited the LPS-induced cytokines expression through downregulating the JAK/STATs signaling in C57/BL6 mice (5 mg/kg, i.p.) and RAW264.7 cells (5, 10, and 20 μM). Daphnetin also reduced ROS production in these doses (Shen et al., 2017).

Another study showed that the praeruptorins D and E (80 mg/kg, gavage), as pyranocoumarins found in *Kitagawia praeruptora* (Dunn) Pimenov (syn. *Peucedanum praeruptorum*) roots, similar to daphnetin inhibited NF-κB and interconnected inflammatory cytokines (IL-6 and TNF-α) in male BALB/c mice with lung injury induced by intranasal administration of LPS (40 μg/ml) and hydrochloric acid (0.1 N). The total protein level, neutrophils, and cell infiltration in BALF were also reduced at 40 and 80 mg/kg of daphnetin (Yu et al., 2013).

IL-17 as one of the prominent inflammatory cytokines is produced by T lymphocyte helper cells whose production is regulated by retinoic acid-related orphan receptor gamma t (RORγt). Esculetin (20 and 40 mg/kg, i.p.) as a hydroxycoumarin is widely found in *Fraxinus* spp., with the potential of reducing lung injury via inhibiting the RORγt and then the suppression of IL-17 in mice. At the same doses, esculetin also inhibited MAPKs and neutrophils/macrophages entry in mice lung (Lee et al., 2020).

Besides, the protective effects of osthole, as prenylated coumarins purified first from the fruit of *Cnidium monnieri* (L.) Cusson (Apiaceae), were reported in several *in vivo* and *in vitro* studies (Table 1). Reducing the expression of cytokines (IL-1β, IL-6, and TNF-α) and blocking the NF-κB and ERK and Akt signaling pathway are of the critical protective mechanisms of osthole on lung injury (Mo et al., 2014; Tsai et al., 2015; Jin et al., 2018). Also, Shi and coworkers proposed that inhibition of ACE2 and Ag (1–7) depletion in lung tissues are other protective mechanisms of osthole (40 mg/kg, gavage) against the lung injury induced by LPS (Shi et al., 2013). Also, ACE2 has shown ameliorating effects on lung injury complications induced by acid, LPS, and viruses, including SARS coronavirus and influenza (Gu et al., 2016).

Isofraxidin is another hydroxycoumarin, isolated from *Fraxinus* spp., with prominent anti-inflammatory effects, especially pulmonary inflammations induced by influenza virus (Jin et al., 2020; Majnooni et al., 2020). Also, isofraxidin (5, 10, and 15 mg/kg, i.p.) showed improving effects on LPS-induced lung injury via reducing the production of inflammatory cytokines (IL-6 and TNF-α) and prostaglandin E_2_ (PGE2). Consequently, it blocked the secretion of PGE2 in mice serum and BALF, also reduced COX-2 gene expression, and led to further improvement of lung damage (Niu et al., 2015).

Consistently, anomalin (Khan et al., 2019), fraxin (Li W. et al., 2019), psoralidin (Yang H. J. et al., 2011), scoparone (Niu et al., 2014), and umbelliferone (Wang D. et al., 2019) are other coumarinic compounds which have ameliorating effects on lung injury (Table 1).

According to the prominent anti-inflammatory effects of natural coumarins, along with their other pharmacological effects, these compounds can be introduced as one of the new sources of drug discovery for the protection and treatment of lung injury.

### Flavonoids and Other Polyphenol Compounds

Structurally, polyphenols are divided into several categories, including flavonoids (flavonols, flavones, flavanones, flavanols, anthocyanins, and isoflavones), phenolic acids (hydroxybenzoic acid and hydroxycinnamic acids), stilbenes, catechins, tannins, and lignans (Pietta et al., 2003) provided in Figure 2. From the mechanistic point of view, the inhibition of MAPKs cascade, phosphatidylinositol 3-kinase (PI3K)/Akt, Src family kinase-Bruton’s tyrosine kinase-Vav, myeloid differentiation factor 88-TLR4, Nrf2/HO-1, and NF-κB (Yu et al., 2014; Liu S. et al., 2018; Wang et al., 2018; de Oliveira et al., 2019; Tsai et al., 2019; Cao et al., 2020), enzymes involved in the arachidonic acid pathway, inflammatory cytokines, and NF-κB signaling pathway are among the main targets of polyphenols in combating inflammation (Santangelo et al., 2007; Guo et al., 2009). They have also shown to suppress the expression of macrophage inflammatory proteins 1α and 2, IL-1β, IL-6, and TNF-α as inflammatory cytokines (Yang et al., 2018; de Oliveira et al., 2019), decrease ROS production and iNOS expression (Cao et al., 2011; Zhu et al., 2019), decrease SOD activity and MDA levels (Mo et al., 2014), and increase the activity of sirtuin 1 as an antioxidant and anti-inflammatory factor (Wang X. et al., 2020), in *in vitro* and *in vivo* studies at different doses and routes of administration. Resveratrol is a stilbenoid widely found in *Vitis vinifera* L. (Vitaceae) fruits and has shown prominent protective effects on lung injury, induced by various methods such as LPS (Cao et al., 2011; Jiang et al., 2016), hypoxia (Özdemir et al., 2014), sepsis (Kolgazi et al., 2006), staphylococcal enterotoxin B (Rieder et al., 2012), nickel (Cao et al., 2020), methamphetamine (Wang X. et al., 2020), bleomycin (Şener et al., 2007), chest trauma (Torun et al., 2017), and cigarette smoke (Bao et al., 2013). Wang and coworkers showed that resveratrol ameliorated sepsis-induced lung injury after 30 mg/kg dose of i.p. administration. In their study, the level of Nrf-2, HO-1, *p*-Akt, IL-10, SOD, and caspase-3 activities as antioxidant and anti-inflammatory markers increased in lung tissue after treatment by resveratrol. Resveratrol was also able to decrease MIP-2, IL-18, and neutrophil leakage in BALF (Wang et al., 2018).

Flavonoids are another class of polyphenolic compounds whose effects on lung injuries have been extensively studied. Li et al. reported that apigenin C-glycoside, a trihydroxyflavone extracted from *Microcos paniculata* L. (Malvaceae), showed protective effects against LPS-induced lung injury in BALB/c mice at 20 and 40 mg/kg oral doses. In their study, the inhibition of inflammatory cytokines and NF-κB signaling pathway were found as the main mechanisms of apigenin (Li et al., 2018). Daidzein (2, 4, and 8 mg/kg, i.p.), an isoflavone widely found in *Glycine max* (L.) Merr. (soybeans, Fabaceae), and myricetin (10, 20, and 40 mg/kg, i.p.), a hexahydroxyflavone widely found in black tea, inhibited TLR4/MyD88/NF-κB cascade and thereby showed protective effects against LPS-induced lung injury in rats (Feng et al., 2015; Mao and Huang, 2017). Also, naringenin and its inhalation pharmaceutical dosage form improved the LPS-induced lung injury in rats. This trihydroxyflavanone compound showed protective effects at 100 mg/kg oral administration and 3 mg/rat inhalant administration doses via downregulating the PI3K/Akt and MAPKs pathways (Zhao et al., 2017; Yu et al., 2020). Besides, anthocyanins such as malvidin derivatives and cyanidin-3-*O*-glucoside, with similar structures to flavonoids, have also shown protective effects on lung injuries (Liu et al., 2015; Yan et al., 2015). Also, wogonin (Yao et al., 2014), rutin (Yeh et al., 2014), quercetin (Wang J. et al., 2015), luteolin (Liu B. et al., 2018), kaempferol (Chen et al., 2012), isorhamnetin (Chi et al., 2016), hyperin (Hu et al., 2019), hesperetin (Wang N. et al., 2019), fisetin (Hussain et al., 2019), breviscapine (Wang et al., 2013), eriodictyol (Zhu et al., 2015), and cardamonin (Wei et al., 2012) are other flavonoid compounds with protective effects on lung injury through different mechanisms (Table 1). Besides, such aforementioned flavonoids, as kaempferol (Schwarz et al., 2014), quercetin (Yang et al., 2020), and myricetin (Yu et al., 2012), in addition to possessing a protective effect on lung injuries, have shown anticoronavirus effects, which increases the importance of their use in the treatment of COVID-19.

Phenolic acid derivatives such as curcumin, chlorogenic acid, caffeic acid, salidroside, rosmarinic acid, and apocynin are other compounds with protective effects on lung injury with various mechanisms (Yildiz et al., 2008; Zhang et al., 2010; Chu et al., 2012; Suresh et al., 2012; Xu et al., 2014; Zhang et al., 2014). Zhang and colleagues showed that curcumin, isolated from *Curcuma longa* L. (Zingiberaceae), at 200 mg/kg, i.p. dose, protected the LPS-induced lung injury in diabetic rats through suppressing NF-κB pathway (Zhang et al., 2015). Also, chlorogenic acid (50 mg/kg) and rosmarinic acid (5, 10, and 20 mg/kg), found in many herbs, decreased LPS-induced lung injury complications via inhibiting neutrophils and cells infiltration in BALF and downregulating ERK/MAPK pathway and increasing antioxidant activities (Zhang et al., 2010; Chu et al., 2012). Salidroside, isolated from *Rhodiola rosea* L. (Crassulaceae), is another phenolic acid compound that showed the protective effects on lung injury. Salidroside at 10 and 120 mg/kg, i.p., inhibited the expression of proinflammatory cytokines, including IL-6, TNF-α, IL-1β, and transforming growth factor-β1 through suppressing LPS-induced lung injury in mice, and paraquat-induced lung injury in rats, respectively (Guan et al., 2012; Zhang et al., 2014). Also, silibinin, as a flavonolignan mixture found in *Silybum marianum* L, showed a potential effect in blocking STAT pathway and reducing proinflammatory cytokines; thereby it could be a promising agent for the treatment of lung injuries in patients with COVID-19 (Bosch-Barrera et al., 2020). Silymarin, magnolol, thearubigin, gossypol, tannic acid, chicoric acid, and ellagic acid are other polyphenol compounds with protective effects on lung injury. The mechanisms, main natural source, and other related information are presented in Table 1. In summary, silymarin, thearubigin, and chicoric acid via upregulating the Nrf2/HO-1 (Zhao et al., 2015; Ding et al., 2019; Wang X. et al., 2019) and magnolol, tannic acid, gossypol, and ellagic acid through downregulating NF-κB pathways improved lung injury (Yunhe et al., 2012; Liu Z. et al., 2013; Guan et al., 2017; Zhang et al., 2019). In addition, silymarin is an undergoing clinical trial study for the treatment of SARS-CoV-2 lung injury (NCT04394208).

In general, due to the anti-inflammatory and antioxidant effects of polyphenol compounds, as well as their antiviral effects (Table 1), this category of secondary metabolisms of plants has the potential to treat COVID-19 and its complications, including lung injuries. However, the pharmacokinetic parameters of these compounds should be considered (Yu et al., 2020).

### Quinones

Quinones are another class of phytochemicals with an aromatic ring attached to two carbonyl groups in their structure, including anthraquinones, benzoquinones, naphthoquinones, phenanthrenequinones, and polycyclic quinones derivatives (Figure 3). Several investigations showed that the quinones derivatives have demonstrated protective effects on lung injury by various mechanisms. Chen and coworkers reported that a phenanthrenequinone isolated from *Salvia miltiorrhiza* Bunge (Lamiaceae), Tanshinone IIA, suppressed the nucleotide-binding oligomerization domain-like receptors pyrin domain-containing protein 3 (NLRP3), as an inflammatory signaling pathway, at 10 mg/kg i.v. in rats, thereby reducing the oleic acid-induced lung injury (Chen T. et al., 2019). Also, emodin, an anthraquinone found in different laxative plants such as *Rheum rhabarbarum* L. (Polygonaceae), showed protective effects on LPS-lung injury via activating autophagy pathways at 20 mg/kg i.p. in rats (Dong et al., 2019). Shikonin (a naphthoquinone) and thymoquinone (a benzoquinone) are other quinones with protective effects on lung injury (Kanter, 2011; Liang et al., 2013) (Table 1).

### Terpenoids and Saponins

Terpenoids are natural carbohydrate compounds, divided into seven categories based on the number of carbons in their structure (Figure 3), including hemiterpenes (C5), monoterpenes (C10), sesquiterpenes (C15), diterpenes (C20), sesterterpenes (C25), triterpenes (C30), and polyterpenes (>C30). These compounds have shown several biological and pharmacological effects, including antioxidant, anticancer and cytotoxic, anti-inflammation, hypoglycemic, antiviral, and analgesic, antimicrobial, and anti-Alzheimer disease effect (Tholl, 2015; Jaeger and Cuny, 2016). In addition, these compounds have shown protective effects on lung injury with different mechanisms. Eucalyptol, thymol, linalool, eugenol, *p*-cymene, and geraniol, as monoterpenes isolated from the essential oils of various plants, have improved the lung injuries induced by cigarette smoke and LPS (Table 1). In terms of mechanism, these effects are exerted through inhibiting the expression of the anti-inflammatory cytokines and suppressing TLR4/NF-κB and MAPKs pathways, along with decreasing the infiltration of proteins, neutrophils, and cells to BALF. They have also indicated protective effects by reducing the Bax/Bcl-2 ratio and increasing the antioxidant activities (Xie et al., 2012; Huo et al., 2013; Huang et al., 2015; Jiang et al., 2017; Wan et al., 2018; de Lima Gondim et al., 2019). On the other hand, oridonin, a diterpenoid found in *Isodon rubescens* (Hemsl.) H.Hara [syn. *Rabdosia rubescens* (Hemsl.) H.Hara] (Lamiaceae), suppressed the NLRP3 signaling pathway, and NF-κB, as well as activating the Nrf2/HO-1 pathway, and thereby showed protective effects on lung injury at 2.5, 5, and 10 μM in *in vitro* study and 20 and 40 mg/kg, i.p. in rats (Yang et al., 2019). Triptolide (Wang et al., 2014), isoforskolin (Yang W. et al., 2011), carnosol (Tian et al., 2010), and andrographolide (Guan et al., 2013) are other diterpenes that are present in various herbal medicines with protective effects on lung injury via activating antioxidant pathways such as Nrf2/HO-1, as well as inhibiting TLR4/NF-κB, proinflammatory cytokines expression, and MAPKs pathways (Table 1). Also, sesquiterpenes, such as artemisitene isolated from A*rtemisia annua* L. (Asteraceae) at 10 mg/kg, i.p., in mice (Chen et al., 2016), zerumbone, presented in *Zingiber zerumbet* (L.) Roscoe ex Sm. (Zingiberaceae) at 10 μM/kg, i.p. (Leung et al., 2017), costunolide, isolated from *Lactuca sativa* L. (Asteraceae) at 30 mg/kg, i.p., in C57BL/6J mice (Chen Y.-t. et al., 2019), and farnesol, isolated from *Cymbopogon commutatus* (Steud.) Stapf (Poaceae) at 100 mg/kg orally (Qamar and Sultana, 2008), showed protective effects against lung damage by activating Nrf2/HO-1 pathway and inhibiting MAPKs pathway and suppressing TNF-α, IL-6, and IL-1β expression. In addition, parthenolide as *Tanacetum parthenium* sesquiterpene lactones inhibited the cytokine storm in inflammatory conditions. Therefore, it can be a good candidate for clinical trial studies of lung injuries induced by SARS-Cov-2 (Bahrami et al., 2020). On the other hand, triterpenoids such as rubriflordilactones A, betulinic acid, and taraxasterol also have shown ameliorating effects on lung injuries induced by LPS and sepsis (San et al., 2014; Lingaraju et al., 2015; Wang Y.-Y. et al., 2015) (Table 1). Wang and coworkers showed that the rubriflordilactone, isolated from *Schisandra sphenanthera* Rehder & E.H.Wilson (syn. *Schisandra rubriflora* Rehder & E.H.Wilson) (Schisandraceae), improved the LPS-lung injury at 10 nM/kg in rats and 10 nM/ml on mouse lung epithelial cell lines (MLE-15) through increasing the expression of sirtuin 1 and suppressing inflammatory markers expression, including MMP9, iNOS, and IL-6 (Wang Y.-Y. et al., 2015). Saponins are other natural compounds, which are classified in the category of terpenoids. Ginsenoside Rg3 (20 and 30 mg/kg) and Rg1 (40 and 200 mg/kg) are two triterpenes saponins, isolated from *Panax ginseng* C.A.Mey. (Araliaceae), with improving effects on LPS-lung injury in mice. These compounds inhibited the infiltration of neutrophils and proteins and M2 macrophage in BALFs and reduced pulmonary edema. Their main mechanism of action is through the suppression of NF-κB (Bao et al., 2015; Cheng and Li, 2016). Increasing the expression of heat shock protein 70 leads to the inhibition of TLR4/MyD88 pathway. The later mechanism is the main protective mechanism of dioscin against lung injury as a steroidal saponin from *Dioscorea* spp. (Dioscoreaceae) (Zeng et al., 2018). Soyasaponin (Lin et al., 2016), glycyrrhizin (Menegazzi et al., 2008), and sodium aescinate (Menegazzi et al., 2008) are other saponin compounds with improving effects on lung injury (Table 1).

### Miscellaneous Natural Compounds

Geniposide (20, 40, or 80 mg/Kg, i.p., mice), as an iridoid found in *Gardenia jasminoides* J.Ellis (Rubiaceae), improved the LPS-induced lung injury via suppressing the NF-κB and MAPKs (Xiaofeng et al., 2012). Sulforaphane, as an isothiocyanate isolated from *Brassica oleracea* L. (Brassicaceae), activated the Nrf2 pathway and inhibited the PGE2, COX-2, and MMP-2 at 50 mg/kg, i.p., in BALB/c mice, thereby ameliorating LPS-induced lung injury (Qi et al., 2016). Consequently, cannabidiol (Figure 3), as cannabinoid derivative of *Cannabis sativa* L. (Cannabaceae), and polysaccharides of *Houttuynia cordata* Thunb. (Saururaceae) showed similar effects through inhibiting the expression of proinflammatory cytokines (TNF-α and IL-6) and reducing the infiltration of cells and proteins in BALF (Ribeiro et al., 2015; Xu et al., 2015). Also, cannabidiol inhibited the cytokines storm-induced by viral infection at 5 mg/kg in C57BL/6 mice (Khodadadi et al., 2020). Besides, a clinical trial is underway on cannabidiol and its derivatives for the treatment of lung injury in patients with COVID-19 (NCT04467918).

In general, due to the protective effects of the aforementioned phytochemicals on lung injuries, these compounds can be used as a protector and treatment in lung injuries leftover from coronavirus activity, including COVID-19. Given the antiviral effects (especially anticoronavirus) of some of the compounds listed in Table 1, this role could lead researchers to find much more effective multitarget compounds in the treatment of patients with COVID-19 and its complications.

## Conclusion

Since the World Health Organization (WHO) announced the pandemic of COVID-19 disease (March 11, 2020), no effective treatment or vaccine has been introduced to treat this disease. Besides, to eliminate the SARS-CoV-2, conventional medications have either failed or been used taking them in doses higher than their therapeutic index leading to side effects (Ianevski et al., 2020; Sharma et al., 2020). On the other hand, due to their multitarget character, phytochemicals have always been of the options for discovering drug molecules to treat complicated diseases, including viral diseases and their complications. On the other hand, lung injury is the main COVID-19 complication that happens with inflammatory cascades by SARS-CoV-2 (Fakhri et al., 2020b; Merad and Martin, 2020). In the present review, we described the candidate phytochemicals with protective effects on lung injuries induced by various methods, as well as their pharmacological mechanisms (Figure 4). In addition, we showed some phytochemicals possessing protective effects against lung injury, with a focus on cepharanthine, epigoitrin, isofraxidin, osthole, resveratrol, apigenin, kaempferol, myricetin, quercetin, chlorogenic acid, chicoric acid, emodin, thymoquinone, betulinic acid, eucalyptol, oridonin, zerumbone, glycyrrhizin, and sulforaphane and their antiviral activities (Table 1). On the other hand, despite the effectiveness of natural secondary metabolites in combating viral diseases, providing the novel drug delivery systems helps to drawback their pharmacokinetic limitations (Abbaszadeh et al., 2020; Fakhri et al., 2020a). Such reports could pave the way for discovering alternative drugs with anti-CoV effects and the potential in controlling the complication of COVID-19. Additional studies are needed to reveal the precise dysregulated pathways in COVID-19 and clarify the potential effects of phytochemicals on humans.

## Author Contributions

MM and MF contributed to conceptualization; MM, SF, and MF contributed to designing the structure of the paper; MM and SF contributed to software; MM, SF, YS, NK, KS, PM, MG, MF, and JE contributed to drafting the manuscript; and MM, SF, MF, and JE contributed to reviewing and editing the paper.

## Funding

JE gratefully acknowledges funding from CONICYT (PAI/ACADEMIA No. 79160109).

## Conflict of Interest

The authors declare that the research was conducted in the absence of any commercial or financial relationships that could be construed as a potential conflict of interest.
